# Surgical management of spinal intradural metastatic pathologies: a case-based review

**DOI:** 10.1093/jscr/rjae108

**Published:** 2024-06-03

**Authors:** Constantinos Thoma, Zachary K Englander, Georgios Prezerakos

**Affiliations:** Department of General Surgery, The Royal London Hospital, London E1 1FR, United Kingdom; Department of Neurosurgery, Columbia University Medical Center, New York, NY 10032, United States; Victor Horsley Department of Neurosurgery, National Hospital for Neurology and Neurosurgery, Queens Square, London WC1N 3BG, United Kingdom

**Keywords:** intradural spinal metastases, spine surgery, complex spine, neurosurgery

## Abstract

Intradural spinal metastases significantly impair neurological function and quality of life, necessitating multimodal, palliative management to preserve mobility and alleviate pain. The effectiveness of systemic chemotherapy and radiotherapy is limited due to the blood-spinal cord barrier and the tumours’ radioresistance, respectively. This highlights the urgency for alternative treatments given the rapid neurological decline. Surgical intervention becomes crucial, focusing on maximum tumour debulking to enhance disease control, restore ambulation, and palliate symptoms without compromising neurological function. Achieving this involves meticulous preoperative planning and aggressive intraoperative neuromonitoring. Combining surgery with adjuvant therapies may improve local control and potentially delay recurrence. This case-based review emphasizes the surgical considerations and outcomes in two cases of intradural spinal metastases, underscoring the value of surgery in multimodal therapy.

## Introduction

Metastatic intradural spinal pathologies constitute a well-established but infrequent subgroup of oncological spinal workload. Their management poses several diagnostic as well as intraoperative challenges to the treating clinician. This includes determining whether operative treatment is appropriate, as well as balancing the preservation of neurological function with maximizing surgical resection. In this paper, we present two cases to illustrate the clinical rationale between various surgical approaches to metastatic intradural tumours, along with their respective outcomes.

## Case 1

A 57-year-old female presented with a 4-month history of gradually worsening lower back pain, combined with lower limb weakness, leading to an abnormal gait. She also noted urinary incontinence, peri-anal numbness, and sensory disturbances including neurogenic claudication. 4 years prior to this presentation, she underwent an awake craniotomy and gross total resection of a lesion within the left frontal lobe, which was infiltrating the frontal horn of the left lateral ventricle. The histopathological diagnosis was that of an anaplastic oligodendroglioma with WHO grade III characteristics, IDH-mutant, and 1p/19q co-deleted. There was no recurrence of the cranial lesion throughout the previous 4-year period.

MRI of the spine revealed an intradural lesion between L4-S2, occupying the thecal sac in its entirety, with motor and sensory rootlets being either displaced or encased by the tumour (Fig. 2). The lesion demonstrated heterogeneous enhancement on administration of gadolinium. The differential diagnosis included a primary tumour such as an ependymoma, a potential drop metastasis from the cranial oligodendroglioma, or a metastasis from an unknown primary.

**Figure 1:**
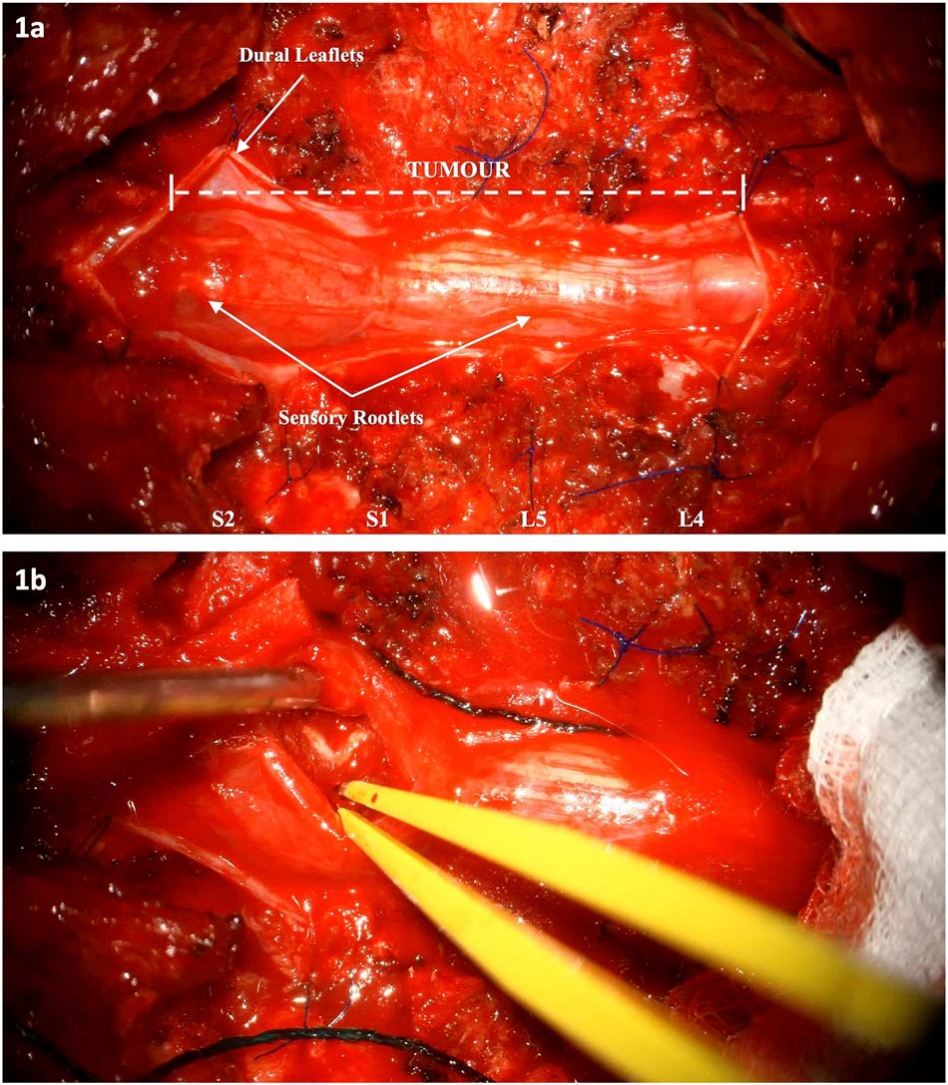
Intraoperative images: (a) Tumour extension from L4 to S2. The dura has been cut and reflected. (b) Internal debulking at the level of S2 whilst respecting the clearly demarcated neural elements. The intrinsic nature of the tumour is noted.

The patient underwent L4-S2 laminectomies with maximal tumour debulking (subtotal resection) and dissection off the nerve roots of the cauda equina (Fig. 1, Video S1). Aggressive intraoperative neuromonitoring was utilized with direct bipolar stimulation. A single rootlet with significant tumour bulk infiltration was sacrificed after confirming no electrophysiological activity. Histopathological testing confirmed the lesion was a drop metastasis from the oligodendroglioma.

**Figure 2:**
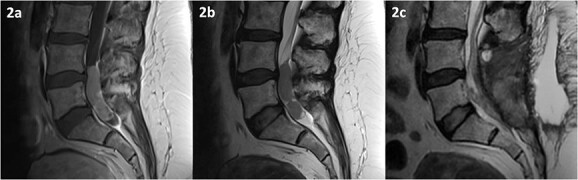
MRI of the spine depicting an intradural lesion between L4-S1. T1-weighted imaging (a) shows heterogeneous enhancement of the lesion upon administration of gadolinium. The lesion is hypointense on T2-weighted imaging (b). Postoperative T2-weighted imaging (c) reveals a small amount of residual tumour.

Postoperatively, the patient exhibited a significant improvement, with complete resolution of her symptomatology. She became fully ambulant and was subsequently discharged 6 days after surgery. Bladder and bowel function were preserved, and there were no signs of perineal sensory impairment. She was seen in clinic 3 weeks after discharge and had no neurological deficits.

## Case 2

A 72-year-old female presented with a 2-month history of lower back pain which radiated bilaterally, along with progressive lower limb weakness. This was accompanied by deteriorating mobility necessitating the use of a Zimmer frame. Neurological examination revealed distal lower limb weakness with right sided foot drop. In addition, reduced anal tone was noted on digital rectal examination. One month prior to this, she had undergone a biopsy for a scalp lesion which was subsequently diagnosed as a poorly differentiated carcinoma.

**Figure 3:**
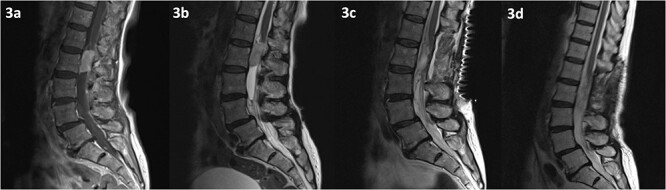
MRI of the spine depicting an intradural lesion at the level of L2, just below the conus. The lesion demonstrates homogenous enhancement on contrast enhanced T1-weighted imaging (a). The lesion is hyperintense on T2-weighted imaging (b). There is no evidence of residual tumour on T2 weighted imaging at 2 days (c) and 14 months (d) postoperatively.

MRI of the spine revealed a 29 × 13 mm intradural soft tissue lesion at the level of L2, just below the conus (Fig. 3). The mass exhibited homogenous enhancement upon the administration of gadolinium. These non-specific specific findings lead to diagnostic uncertainty regarding the nature of the lesion. Nonetheless, there is an urgent need to halt or reverse the ongoing neurological deterioration.

The patient underwent an L1–2 laminectomy with complete piecemeal resection of the tumour. During the operation, a haemorrhagic, multiloculated tumour was identified, intertwined with several nerve roots. One nerve root, infiltrated by the tumour, had to be sacrificed for complete tumour resection. This was deemed non-functioning through electrophysiology. Histopathological examination revealed a metastatic renal cell carcinoma (RCC).

Postoperatively, the patient improved significantly. Despite persistent lower limb weakness, she underwent intensive physiotherapy and was able to mobilize independently with the assistance of walking aids. She was subsequently discharged 1 month after surgery. There was no evidence of recurrence 14 months postoperatively. Sixteen months after the operation, she was reviewed in clinic and was noted to be independent in walking.

## Discussion

Intradural spinal metastases are rare, making up ~0.8–3.9% of symptomatic metastases affecting the spinal cord and observed in only 2% of patients who die from systemic cancer [1, 2]. Simultaneously, spinal drop metastases from an intracranial pathology are thought to occur in 1–3% of cases, however the true incidence rate is unknown [1, 3]. Peak incidence of spinal intradural metastases is between 40 and 65 years of age, which corresponds to the period of highest cancer risk [4, 5]. Clinically, patients most often present with lower back pain and radicular pain, which occurs in 83–95% of cases [4]. In addition, one series notes that sensory and motor deficits occur in 58 and 54% of cases, respectively. The same series found that acute cauda equina syndrome is observed in approximately one-third of cases [6].

The understanding of how metastatic cells reach the intradural space is continually refined with emerging case studies and reports. Presently, five predominant mechanisms have been identified. These include haematogenous spread via the arterial system, retrograde spread through Batson’s venous plexus, local invasion, spread via perineural lymphatics, and cerebrospinal fluid (CSF) seeding in the so call ‘drop metastasis’ fashion [7]. Capek et al. suggested that RCC may metastasize to the intradural space via perineural spread, propagating along the peripheral nervous system [8]. In our cases, the metastatic mechanism of Case 1 is likely through CSF seeding, a hypothesis further supported by the patient’s history of prior cranial surgery and the wide opening of the left frontal horn during the surgical resection. We cannot say with certainty the mechanism of metastasis of Case 2.

Metastatic spinal intradural pathologies pose not only diagnostic difficulties, but also present with challenges as to their intraoperative management. In most cases, conducting a biopsy is not feasible due to factors such as tumour location, the patient’s performance status, and the risk of neurological damage and CSF leak [9]. Thus, reliance on imaging findings and patient history becomes crucial in determining the nature of the lesion. From our experience, there are instances where the spinal lesion is the only one present, even in patients with a history of malignancy, particularly those in long remission. Therefore, in such cases, a metastatic intradural pathology is usually not the most likely diagnosis, as primary lesions like ependymomas take precedence.

The management of intradural spinal metastases presents a complex therapeutic challenge, particularly when considering the limitations of systemic treatments. Systemic chemotherapy, often the cornerstone of oncological management, faces significant limitations in this context due to the restrictive nature of the blood-spinal cord barrier and the frequent uncertainty surrounding the histological nature of the spinal lesions. This uncertainty hampers the ability to tailor chemotherapy effectively to the specific tumour type. Similarly, the role of radiotherapy is constrained, especially in cases involving non-radiosensitive tumours, such as RCC. Moreover, the acute nature of neurological decline in patients with spinal metastases often necessitates a more immediate intervention, rendering these options less suitable [10–12]. These challenges underscore the necessity of incorporating surgical intervention into the treatment paradigm.

Striving for maximal debulking without compromising neurological function remains the best feasible goal, and employing aggressive electrophysiological monitoring can aid in achieving this objective. Seeking complete total resection of the lesion can potentially lead to neurological deficits. Considering the palliative nature of the operation for metastatic disease, where the goal is to decompress the neural structures and enhance local control rather than achieve a cure, the justification for this approach may be questioned. En bloc resection can be considered for isolated, well demarcated metastases. However, this approach can be technically challenging and riskier in terms of potential damage to surrounding structures. Therefore, our surgical approach prioritizes maximal tumour debulking while meticulously preserving neurological function, aligning with the primary goal of improving patient quality of life and managing symptoms effectively. Supplementary intraoperative techniques such as ultrasonography can also be implemented to assist in deciding the extent of laminectomy, durotomy and resection of the lesion [13].

Alternatively, palliative techniques like expansion duroplasty can be considered, providing sufficient space for symptom management without sacrificing neurological function. However, the drawback of this approach is that the tumour is left behind. Nonetheless, when combined with systemic treatments, such an approach may improve quality of life for an extended period [14].

Surgery not only addresses the immediate need to manage neurological decline, but also provides a path to obtain a definitive histological diagnosis. This diagnosis is crucial for guiding subsequent adjuvant therapies. Postoperative chemotherapy and radiation can be particularly effective in targeting residual tumour cells, enhancing local control, and potentially delaying or preventing recurrence. The integration of these therapies with surgical intervention forms a comprehensive treatment strategy, aiming to improve patient outcomes and quality of life [15].

Finally, postoperative care with focused spinal nursing and physical rehabilitation is equally paramount, playing a pivotal role in guiding patients towards the restoration of their mobility and the reestablishment of their baseline function [16]. This cooperative approach is instrumental in ensuring a successful recovery journey, ultimately improving patients’ overall well-being and quality of life.

## Conclusion

In essence, this review emphasizes that surgery can be an integral element of multimodal therapy and contributes significantly to the comprehensive management of spinal intradural metastatic pathologies. It should be considered in select cases as it serves as a powerful tool in preventing neurological deterioration, improving mobility, and ultimately increasing the quality of life for patients.

## Supplementary Material

Video_1_rjae108
